# Residual Negative Symptoms Differentiate Cognitive Performance in Clinically Stable Patients with Schizophrenia and Bipolar Disorder

**DOI:** 10.1155/2014/785310

**Published:** 2014-06-12

**Authors:** Rajeev Krishnadas, Seethalakshmi Ramanathan, Eugene Wong, Ajita Nayak, Brian Moore

**Affiliations:** ^1^Sackler Institute of Psychobiological Research, Southern General Hospital, Room 25, University Corridor, Ground Floor, Neurology Building, Glasgow G51 4TF, UK; ^2^Hutchings Psychiatric Center, NYS Office of Mental Health, Syracuse, NY 13210, USA; ^3^Gartnavel Royal Hospital, Glasgow G12 0XH, UK; ^4^Department of Psychiatry, KEM Hospital, Mumbai 400012, India; ^5^Newcastle General Hospital, 1st Floor, Building 15, Westgate Road, Newcastle upon Tyne NE4 6BE, UK

## Abstract

Cognitive deficits in various domains have been shown in patients with bipolar disorder and schizophrenia. The purpose of the present study was to examine if residual psychopathology explained the difference in cognitive function between clinically stable patients with schizophrenia and bipolar disorder. We compared the performance on tests of attention, visual and verbal memory, and executive function of 25 patients with schizophrenia in remission and 25 euthymic bipolar disorder patients with that of 25 healthy controls. Mediation analysis was used to see if residual psychopathology could explain the difference in cognitive function between the patient groups. Both patient groups performed significantly worse than healthy controls on most cognitive tests. Patients with bipolar disorder displayed cognitive deficits that were milder but qualitatively similar to those of patients with schizophrenia. Residual negative symptoms mediated the difference in performance on cognitive tests between the two groups. Neither residual general psychotic symptoms nor greater antipsychotic doses explained this relationship. The shared variance explained by the residual negative and cognitive deficits that the difference between patient groups may be explained by greater frontal cortical neurophysiological deficits in patients with schizophrenia, compared to bipolar disorder. Further longitudinal work may provide insight into pathophysiological mechanisms that underlie these deficits.

## 1. Introduction

Cognitive deficits represent stable traits in both schizophrenia and bipolar disorder [1]. Studies that have directly compared the two groups show qualitatively similar deficits, but quantitatively, milder deficits in bipolar disorder [2–4]. More recently, it has been postulated that this quantitative difference may depend on the presence or severity of psychotic symptoms [5, 6]. For example, Simonsen et al. found that as compared to those without psychosis, subjects with a history of psychosis, irrespective of the diagnosis, showed poorer performance on neurocognitive measures [5]. Additionally, depressive and negative symptoms have also been associated with cognitive deficits [7, 8]. However, very few studies have tried to address these issues during symptomatic remission. It is not clear if residual/subthreshold psychopathology during periods of remission would explain the trait difference between the two groups. In other words, do patients with schizophrenia perform poorer on cognitive tests than patients with bipolar disorder, due to the presence of residual/subthreshold psychotic/negative symptoms?

In this study, we aimed to compare cognitive function in patients with euthymic bipolar disorder with those with schizophrenia in remission. Our second aim was to examine if differences in residual symptoms (psychotic/negative symptoms) between the patient groups could explain (mediate) the difference in cognitive dysfunction between the groups. In other words, the question we are attempting to answer is whether the presence of residual symptoms, including psychotic and specifically negative symptoms, in schizophrenia explains the poorer cognitive performance in schizophrenia? We hypothesise that the difference in cognitive performance could be explained by the presence of residual symptoms.

## 2. Materials and Methods

The study received ethical approval from the institutional ethics review board, and all subjects gave written informed consent. Twenty-five 18–60 year-old subjects were recruited into each group. Both the patient groups were recruited from the outpatients' clinics of BYL Nair Hospital. Those in the bipolar disorder group (BD), fulfilled the DSM IV criteria for bipolar I disorder—most recent episode manic—severe without psychotic features (with no life-time history of a psychotic episode confirmed using MINI 5.0). Those in the schizophrenia group (SZ), fulfilled the DSM IV criteria for schizophrenia [10, 11]. Euthymia was ascertained using a cut-off score of less than 8 on Hamilton Depression Rating Scale (HRSD) and Young Mania Rating Scale (YMRS) [12, 13]. Remission in schizophrenia was confirmed using a cut-off score of 8 with a score of less than 2 on individual items on the Brief Psychiatric Rating Scale (BPRS) and Scale for the Assessment of Negative Symptoms (SANS) [9, 14, 15]. Antipsychotic doses were measured using the defined daily dose (DDD) method described by WHO [16]. The control group consisted of 25 healthy subjects who had no past or family history of major psychiatric illness in a first-degree relative. Subjects with comorbid Axis I (including substance use disorder—except nicotine use based on MINI 5.0 screening) and Axis II diagnoses, those with medical comorbidity, and those who had received ECT in the last 6 months were excluded.

### 2.1. Cognitive Assessment

#### 2.1.1. Verbal and Visual Memory

Memory was assessed using Postgraduate Institute Memory Scale (PGIMS), an Indian adaptation of Wechsler memory scale and Boston memory scale that takes into account the language, educational level, and norms standardized for the Indian population [17]. Dysfunction scores adjusted for education level are scored 0, 2, or 3, with higher scores suggesting greater dysfunction. We used the total dysfunction score as well as the raw scores on digit span tests. The Rey-Osterrieth complex figure test with copy component (ROCFT1) and a 30-minute recall component (ROCFT2) was used to evaluate visual attention (copy component), visuoconstructional ability, and delayed visual memory [18].

#### 2.1.2. Trail-Making Test (TMT)

TMT A and B have been commonly used and validated in the Indian population [19].

#### 2.1.3. Verbal Fluency

Based on previous studies of verbal fluency, one Hindi phoneme (denoted in English as* /p/*[“pa:”]) was used in this task and participants were asked to generate words that started with the sounds associated with these letters in the 60 s time limit. The total number of words in the trial, after excluding proper nouns, numbers, and multiple forms of the same root word, was taken as the verbal fluency score [20].

#### 2.1.4. Go-No Go Task

Sensitivity to interference and inhibitory control was tested using a two-step go-no go task, as described in the frontal assessment battery [21]. In the first task, the subject was asked to provide an opposite response to the examiner's alternating signal. Thus, the subject should obey the initial verbal command and refrain following what they see. On the second task, the subject must inhibit a response that was previously given to the same stimulus. A total of the correct responses on the two tests were taken as the total score.

## 3. Statistical Analysis

Firstly, we examined zero-order correlation between demographic/clinical variables and cognition (b path on mediation). To address our first aim, we compared cognitive function between groups using MANCOVA, with cognitive functions as dependent variable, diagnosis as the fixed factor, and those variables that correlated with cognitive function (dependent variable), but not diagnostic group (independent variable), as covariates [22]. We correlated the effect sizes of cognitive deficits between the two diagnostic groups to look at the similarity of cognitive deficits, as previously done by Schretlen et al. [3].

**Figure 1:**
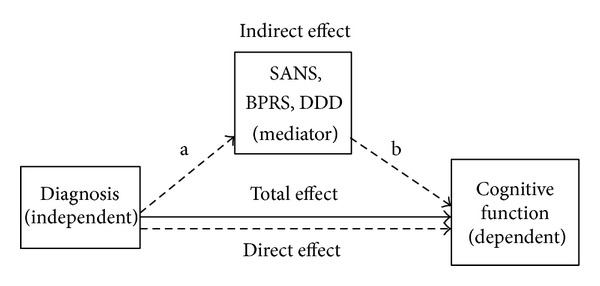
Mediation analysis. The figure depicts the relationship between the independent, mediator, and dependent variables. The mediation analysis partitions the total variance (total effect) explained by the predictor into a part that is independent of the mediating variable (direct effect) and a part that is accounted for via the mediating variable (indirect effect). a represents the “a” path and b represents the “b” path.

Finally, to address our second aim, we conducted a mediation analysis to see if variables, SANS, BPRS, and DDD (mediators), that differed significantly between diagnostic groups mediated the relationship between group membership (independent variable: IV) and cognitive function (dependent variable: DV) in each domain. The analysis tests the hypothesis that the diagnostic category (IV) accounts for variance in the mediator and in turn, this variance in the mediator accounts for a proportion of the variance of the cognitive deficits (DV) (Figure 1). “The adjustable parameters of the model represent the unidirectional influence between pairs of variable in the model. The best fitting values of the parameters are estimated by using the General Linear Model to solve the linear equations that describe the relationships within the model. This analysis differs from multiple regression which estimates the proportion of variance in the dependent variable accounted for by each of several independent predictor variables while allowing for the variance accounted for by the other predictors in the model. In other words, the mediation analysis partitions the variance explained by the predictor into a part that is independent of the mediating variable and a part that is accounted for via the mediating variable” [23]. In classic mediation analysis, causal models are tested using longitudinal data; such an assumption was not made here. The purpose of this analysis was to identify overlapping variance in cognitive function, explained by the diagnostic groups and mediator variables. This type of approach has been taken previously to examine the contribution of MRI surface based measures to volume-based measures measured using VBM [19]. We used the bootstrap method of Preacher and Hayes to estimate the indirect effect and bias-corrected 95% confidence interval (CI) for each mediator based on 20,000 bootstrap samples using an SPSS macro [24]. This analysis requires no assumption regarding the underlying distributions since the statistical significance level is determined nonparametrically.

## 4. Results

Demographic and illness variables are shown in Table 1. The two patient groups did not differ on most of the variables. Notably, BPRS, SANS scores, and defined daily dose (DDD) of antipsychotics were greater in the schizophrenia group compared to the bipolar group.

The results of the correlation are shown in Table 2. Age showed a significant association with performance on trail-making test. HDRS scores were associated with memory dysfunction. DDD of medication was associated with poorer performance on digit forward trail-making test B and ROCFT. There was also a significant association between BPRS and SANS across a number of domains.

**Figure 2:**
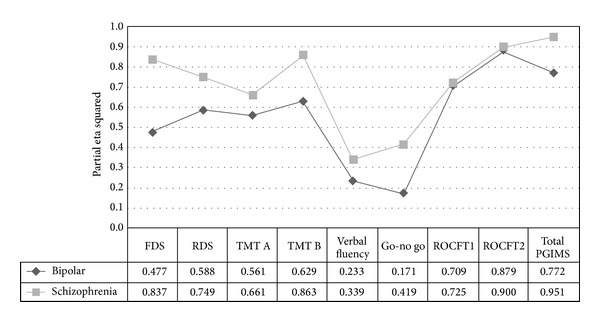
Comparison of effect sizes of differences between cases and controls. Partial eta squared represents the size of the relationship between diagnosis and cognitive function. Greater values represent greater cognitive deficits. Covariates appearing in the model are evaluated at the following values: gender = 0.3067, number of years in education = 9.69, and age = 37.03. FDS: forward digit span; RDS: reverse digit span; TMT A: trail-making test A; TMT B: trail-making test B; ROCFT-ROCFT: Rey Osterreith Complex figure test; PGIMS: PGI memory scale.

Mean effect size of cognitive deficits was *η*_*p*_^2^ = −0.56 in bipolar disorder and *η*_*p*_^2^ = −0.72 in schizophrenia.  Figure 2 shows the relationship between the effect sizes of cognitive function in both groups. As can be seen, patients performed worse than controls on all tests, after controlling for gender, age, and number of years in education. Performing a Pearson's correlation of effect sizes between groups tested the similarity of cognitive deficits. This was found to be *r* = 0.88.

Results of MANCOVA comparing the patient groups are shown in Table 3. Diagnosis was significantly associated with cognitive performance [Pillai's trace = 0.73; *F*(9, 35) = 10.41; *P* < 0.001; *η*_*p*_^2^ = 0.73]. SANS, BPRS, and DDD were not included as covariates in the above model, as they differed significantly between the patient groups. They were considered as potential mediators.

On mediation analysis, we found that SANS mediated the difference in performance between the two groups on trail-making B (Beta = −30.46; *Z* = −3.07; 95%CI = −49.67–−6.52), verbal fluency (Beta = −2.24; *Z* = −2.65; 95%CI = −4.16–−0.18), go-no go test (Beta = −1.59; *Z* = −3.97; 95%CI = −2.615–−0.61), and ROCFT2 (Beta = 2.45; *Z* = 2.09; 95%CI = 0.17–4.89). BPRS and DDD showed no mediation effects.

## 5. Discussion

We have shown that clinically stable patients with schizophrenia and bipolar disorder have qualitatively similar cognitive deficits. These deficits are quantitatively greater in patients with schizophrenia. This difference in performance between the patients groups was at least in part mediated by residual negative symptoms.

Recent review of meta-analyses of cognitive function in patients with remitted bipolar disorder and schizophrenia showed a weighted effect size in the range of 0.6 and 0.8–1 for letter fluency; 0.7 and 0.9 on trail-making tests; 0.7 and 0.9 on digit span backwards; 0.8 and 1.3 on verbal learning and recall, respectively [1]. Findings of our study confirm the large effect sizes observed by the aforementioned investigators in a sample from a developing country. The effect sizes of cognitive deficits in bipolar disorder and schizophrenia showed a high degree of correlation, suggesting deficits in qualitatively similar cognitive function. Schretlen et al. previously compared cognitive functioning in bipolar disorder (*d* = −0.59) with schizophrenia (*d* = −0.97) [3]. They found good correlation (*r* = 0.71) of effect sizes of cognitive tests between the two illnesses, a finding replicated in our study (*r* = 0.88). Similar results have also been shown by studies by Siedman et al. (*r* = 0.81) and Dickerson et al. (*r* = 0.93) [25, 26].

Most interestingly, using mediation analysis, we found that residual negative symptoms mediated the relationship between diagnosis and cognitive function, particularly on the executive function domain. This implies that negative symptoms and cognitive symptoms share significant common variance. In other words, poorer performance on cognitive tests by patients with schizophrenia is, at least in part, explained by the presence of residual negative symptoms in patients with schizophrenia. Neither residual BPRS scores nor greater DDD explained this relationship. This finding is in contrast to those of Simonsen et al., who found that neurocognitive dysfunction was more strongly determined by a history of psychosis [5].

Data from longitudinal studies examining the relationship between negative symptoms and cognitive dysfunction have demonstrated a temporal link between the two. Nevertheless, cognitive and negative symptoms have been shown to be sufficiently independent of each other, implying differential involvement of brain regions in these two processes [7]. More recently, Farzan et al. examined cortical inhibition of gamma band oscillations (CI_*γ*_) in patients with schizophrenia and bipolar disorder. They found that patients with schizophrenia demonstrated selective deficits in CI_*γ*_ in the DLPFC compared to bipolar disorder and healthy subjects. They suggest that this lack of inhibition of gamma oscillations in the dorsolateral prefrontal cortex may represent a frontal neurophysiological deficit that could be responsible for the spectrum of frontal deficits in patients with schizophrenia, including executive and cognitive control deficits and negative symptoms [27, 28]. While we cannot make conclusions about the pathophysiology of these illnesses, our findings may be explained by the greater deficits in these physiological processes in patients with schizophrenia, compared to those with bipolar disorder.

While the positive aspect of our study includes the use of standardised tests in a clinical sample free of substance use and other comorbidities, the study has a number of limitations. The small sample size might have led to a type-2 error. However, the observation that negative symptoms (SANS) mediated the difference in cognitive function, despite the small sample size, suggests that the relationship is robust. It should also be noted that the bias-corrected bootstrapping method for testing the mediation is relatively robust against the effect of small sample sizes, especially when the effect sizes of the “a” path and “b” path of the mediation are large as in our sample. A possible explanation for the finding in our study is that SANS designates a number of occupational and educational performance deficits as negative symptoms that are linked with poor cognitive function [7]. It could be argued that BPRS is a general measure of psychopathology, rather than just a measure of psychosis. However, in our sample, we found that BPRS scores did not correlate significantly with any of the other psychopathology scores in patients with schizophrenia. We did not test the premorbid IQ of the patients. Previous studies have indeed shown that the difference in premorbid IQ could explain some of the differences in performance on verbal fluency and response inhibition tests [29]. Premorbid IQ has also been shown previously to link to negative symptoms. However, previous validating studies have shown that scores on PGIMS show low correlation with IQ [17]. The present study did not confirm remission prospectively. While we excluded those with a history of substance use disorder or recent substance use, this exclusion was based on self-report (and MINI screen). We did not use laboratory methods to rule out recent substance use that may have influenced performance on the cognitive tests. Finally, it is not known how generalizable the data is to the community, where symptoms might not be as controlled as in the population tested.

## 6. Conclusions

Persistent cognitive deficits are seen in patients with euthymic bipolar disorder and schizophrenia under remission when compared to healthy controls. The cognitive deficits are qualitatively similar but worse in patients with schizophrenia compared to patients with bipolar disorder. The worse cognitive function in patients with schizophrenia was mediated by the presence of residual negative symptoms. Further work exploring the link between negative symptoms, cognitive symptoms, and neurophysiological measures may provide insight into pathophysiological mechanisms that may underlie these deficits.

## Figures and Tables

**Table 1:** Demographic and illness characteristics.

	BP	SZ	HC	Test statistic	*P*	BP versus SZ
Female, *n* (%)	7 (28)	9 (36)	7 (28)	Chi square = 0.502	0.778	
Age, years : mean (s.d.)	35.44 (11.18)	40.16 (8.153)	35.48 (5.49)	*F* = 2.49^b^	0.09	
Years in education	10.08 (2.08)	9.08 (1.47)	9.92 (1.03)	*F* = 2.86^b^	0.064	
Unemployed	3	6	0	2.7*E* − 09^a^	<0.001	NS
YMRS, mean (s.d.)	2.28 (1.1)	2.04 (1.02)	0	*F* = 52.32^b^	<0.001	NS
HRSD, mean (s.d.)	1.52 (0.58)	1.36 (0.95)	0	*F* = 41.85^b^	<0.001	NS
BPRS, mean (s.d.)	0.56 (0.65)	3.68 (1.24)	0	*F* = 148.67^b^	<0.001	*t* = −11.07; *P* < 0.001
SANS, mean (s.d.)	0.32 (0.55)	2.68 (0.69)	0	*F* = 204.2^b^	<0.001	*t* = −13.3; *P* < 0.001
IDEAS, mean (s.d.)	1.52 (2.00)	2.16 (2.26)	—	*F* = 1.12^b^	0.295	
Number of manic episodes, mean (s.d.)	2.88 (1.45)	—	—			
Number of depressive episodes, mean (s.d.)	1.08 (1.57)	—	—			
Duration of illness, mean (s.d.)	8.48 (6.61)	11.32 (5.81)	—	*F* = 2.6^b^	0.113	
Medication						
DDD	0.72 (0.50)	1.68 (0.62)		*t* = −5.94;	<0.001	
Lithium, *n* (%)	7 (28)	—	—			
Valproate, *n* (%)	6 (24)	—	—			
Carbamazepine, *n* (%)	6 (24)	—	—			
Lithium + carbamazepine, *n* (%)	6 (24)	—	—			
Antipsychotics, *n* (%)	18 (72)	25 (100)	—			
Benzodiazepine, *n* (%)	5 (20)	8 (32)	—			
Anticholinergics, *n* (%)	8 (32)	24 (96)	—			
Antidepressant, *n* (%)	3 (12)	3 (12)	—			

^a^Fisher's exact test for rXc tables; ^b^ANOVA IDEAS: Indian Disability Evaluation and Assessment Scale; DDD: defined daily dose of antipsychotics; BP: bipolar disorder; SZ: schizophrenia; HC: healthy control; BPRS: Brief Psychiatric Rating Scale (measured on a scale of 0–6 as in [9]); HDRS: Hamilton Depression Rating Scale; YMRS: Young Mania Rating Scale; SANS: Scale for the Assessment of Negative Symptoms.

**Table 2:** Spearman's correlation between clinical and demographic variables and performance on cognitive tests.

	Digit forward	Digit backward	Trail making A	Trail making B	Verbal fluency	Go-no go	ROCFT1	ROCFT2	PGIMS
Age	−0.164	−0.032	−0.313∗	0.263	−0.247	−0.183	0.075	−0.187	0.191
Number of years in education	0.119	0.069	−0.045	−0.156	0.235	0.031	0.127	0.268	0.066
Duration of illness in years	−0.082	0.256	−0.079	0.096	−0.122	−0.132	0.123	−0.215	0.047
DDD	−0.362∗∗	−0.186	0.146	0.433∗∗	−0.245	−0.160	−0.016	−0.500∗∗	0.483∗∗
YMRS	0.137	0.191	−0.175	−0.151	0.154	0.169	−0.052	0.040	−0.056
HDRS	0.213	−0.010	0.244	−0.126	−0.186	−0.154	0.094	0.170	−0.300∗
BPRS	−0.617∗∗	−0.282∗	0.172	0.575∗∗	−0.478∗∗	−0.298∗	0.097	−0.589∗∗	0.558∗∗
SANS	−0.526∗∗	−0.239	0.157	0.466∗∗	−0.488∗∗	−0.511∗∗	0.120	−0.530∗∗	0.526∗∗
Total IDEAS	−0.374∗∗	−0.416∗∗	−0.048	0.131	−0.119	−0.331∗	0.054	−0.117	0.434∗∗

*P < 0.05; **P < 0.01; DDD: defined daily dose of antipsychotics; BPRS: Brief Psychiatric Rating Scale; HDRS: Hamilton Depression Rating Scale; YMRS: Young Mania Rating Scale; SANS: Scale for the Assessment of Negative Symptoms; IDEAS: Indian Disability Evaluation and Assessment Scale.

**Table 3:** Comparison of cognitive performance between patient groups.

	Bipolar^a^	Schizophrenia^a^	*F*	Sig
Total PGIMS	11.26^a^	17.94^a^	33.735	<0.001
Digit span forward	4.75^a^	3.57^a^	26.771	<0.001
Digit span backward	2.84^a^	2.32^a^	5.011	0.030
ROCFT 1	31.57^a^	31.71^a^	0.094	0.761
ROCFT 2	18.44^a^	14.55^a^	31.243	<0.001
Trail-making A	70.81^a^	78.99^a^	1.761	0.192
Trail-making B	118.99^a^	149.37^a^	24.107	<0.001
Verbal fluency test	13.06^a^	11.82^a^	5.371	0.025
Go-no go test	5.52^a^	4.84^a^	6.694	0.013

^a^Estimated marginal means; covariates appearing in the model are evaluated at the following values: age = 37.80, gender = 0.3200, number of years in education = 9.58, hdrs = 1.44, ymrs = 2.16. Adjustment for multiple comparisons: Bonferroni. ROCFT: Rey Osterreith Complex figure test; PGIMS: PGI memory scale.
